# *Plasmodium* infection is associated with cross-reactive antibodies to carbohydrate epitopes on the SARS-CoV-2 Spike protein

**DOI:** 10.1038/s41598-022-26709-7

**Published:** 2022-12-22

**Authors:** Sarah Lapidus, Feimei Liu, Arnau Casanovas-Massana, Yile Dai, John D. Huck, Carolina Lucas, Jon Klein, Renata B. Filler, Madison S. Strine, Mouhamad Sy, Awa B. Deme, Aida S. Badiane, Baba Dieye, Ibrahima Mbaye Ndiaye, Younous Diedhiou, Amadou Moctar Mbaye, Cheikh Tidiane Diagne, Inés Vigan-Womas, Alassane Mbengue, Bacary D. Sadio, Moussa M. Diagne, Adam J. Moore, Khadidiatou Mangou, Fatoumata Diallo, Seynabou D. Sene, Mariama N. Pouye, Rokhaya Faye, Babacar Diouf, Nivison Nery, Federico Costa, Mitermayer G. Reis, M. Catherine Muenker, Daniel Z. Hodson, Yannick Mbarga, Ben Z. Katz, Jason R. Andrews, Melissa Campbell, Ariktha Srivathsan, Kathy Kamath, Elisabeth Baum-Jones, Ousmane Faye, Amadou Alpha Sall, Juan Carlos Quintero Vélez, Michael Cappello, Michael Wilson, Choukri Ben-Mamoun, Richard Tedder, Myra McClure, Peter Cherepanov, Fabrice A. Somé, Roch K. Dabiré, Carole Else Eboumbou Moukoko, Jean Bosco Ouédraogo, Yap Boum, John Shon, Daouda Ndiaye, Adam Wisnewski, Sunil Parikh, Akiko Iwasaki, Craig B. Wilen, Albert I. Ko, Aaron M. Ring, Amy K. Bei

**Affiliations:** 1 Department of Epidemiology of Microbial Diseases, Yale School of Public Health, New Haven, CT 06510 USA; 2 Department of Immunobiology, Yale School of Medicine, New Haven, CT 06510 USA; 3 Department of Laboratory Medicine, Yale School of Medicine, New Haven, CT 06510 USA; 4 Laboratory of Parasitology and Mycology, Aristide le Dantec Hospital, Cheikh Anta Diop University, Dakar, Senegal; 5 DiaTROPIX Rapid Diagnostic Tests Facility, Institut Pasteur de Dakar, Dakar, Senegal; 6 Pôle Immunophysiopathologie et Maladies Infectieuses, Institut Pasteur de Dakar, Dakar, Senegal; 7 G4-Malaria Experimental Genetic Approaches and Vaccines, Pôle Immunophysiopathologie et Maladies Infectieuses, Institut Pasteur de Dakar, Dakar, Senegal; 8 Pôle Virologie, Institut Pasteur de Dakar, Dakar, Senegal; 9 Instituto de Saúde Coletiva, Universidade Federal da Bahia, Salvador, BA Brazil; 10 Instituto Gonçalo Moniz, Fundação Oswaldo Cruz, Ministério da Saúde, Salvador, BA Brazil; 11 Faculty of Medicine, Federal University of Bahia, Salvador, Brazil; 12 Douala Military Hospital, Douala, Cameroon; 13 Division of Infectious Diseases, Department of Pediatrics, Northwestern University Feinberg School of Medicine, Ann & Robert H. Lurie Children’s Hospital of Chicago, Chicago, USA; 14 Division of Infectious Diseases and Geographic Medicine, School of Medicine, Stanford University, Stanford, CA USA; 15 Yale Center for Clinical Investigation, Yale School of Medicine, New Haven, CT USA; 16 Serimmune, Inc., Goleta, CA USA; 17 Grupo de Investigación Ciencias Veterinarias Centauro, University of Antioquia, Medellín, Colombia; 18 Grupo de Investigación Microbiología Básica y Aplicada, University of Antioquia, Medellín, Colombia; 19 Department of Pediatrics, Yale School of Medicine, New Haven, CT USA; 20 Department of Parasitology, Noguchi Memorial Institute for Medical Research, University of Ghana, Accra, Ghana; 21 Department of Internal Medicine, Section of Infectious Diseases, Yale School of Medicine, New Haven, CT USA; 22 Institut de Recherche en Sciences de La Santé (IRSS)/Centre Muraz, Bobo-Dioulasso, Burkina Faso; 23 Department of Biological Sciences, Faculty of Medicine and Pharmaceutical Sciences, University of Douala, Douala, 2701 BP Cameroon; 24 Malaria Research Unit, Center Pasteur Cameroon, Yaoundé, Cameroon; 25 Médecins Sans Frontières, University of Yaoundé and Epicentre, Yaoundé, Cameroon; 26 Department of Internal Medicine, Yale Occupational and Environmental Medicine Program, Yale School of Medicine, New Haven, CT USA; 27 Department of Infectious Disease, Imperial College London, St Mary’s Campus, London, W2 1PG UK; 28 Chromatin Structure and Mobile DNA Laboratory, The Francis Crick Institute, London, NW1 1AT UK; 29 Crick COVID19 Consortium, Francis Crick Institute, London, NW1 1AT UK; 30 Howard Hughes Medical Institute, Chevy Chase, MD USA; 31 Present Address: South London Specialist Virology Centre, Kings College Hospital NHS Foundation Trust, London, UK

**Keywords:** Infectious-disease diagnostics, Malaria, Viral infection

## Abstract

Sero-surveillance can monitor and project disease burden and risk. However, SARS-CoV-2 antibody test results can produce false positive results, limiting their efficacy as a sero-surveillance tool. False positive SARS-CoV-2 antibody results are associated with malaria exposure, and understanding this association is essential to interpret sero-surveillance results from malaria-endemic countries. Here, pre-pandemic samples from eight malaria endemic and non-endemic countries and four continents were tested by ELISA to measure SARS-CoV-2 Spike S1 subunit reactivity. Individuals with acute malaria infection generated substantial SARS-CoV-2 reactivity. Cross-reactivity was not associated with reactivity to other human coronaviruses or other SARS-CoV-2 proteins, as measured by peptide and protein arrays. ELISAs with deglycosylated and desialated Spike S1 subunits revealed that cross-reactive antibodies target sialic acid on N-linked glycans of the Spike protein. The functional activity of cross-reactive antibodies measured by neutralization assays showed that cross-reactive antibodies did not neutralize SARS-CoV-2 in vitro. Since routine use of glycosylated or sialated assays could result in false positive SARS-CoV-2 antibody results in malaria endemic regions, which could overestimate exposure and population-level immunity, we explored methods to increase specificity by reducing cross-reactivity. Overestimating population-level exposure to SARS-CoV-2 could lead to underestimates of risk of continued COVID-19 transmission in sub-Saharan Africa.

## Introduction

Serological surveillance studies provide a fundamental understanding of past exposure to infectious diseases such as SARS-CoV-2. SARS-CoV-2 infection induces immune responses to both Spike and Nucleocapsid proteins, which have been used in serologic tests to determine exposure history^1^. Serological surveillance for SARS-CoV-2 has been used in Africa to estimate exposure with seropositivity estimates ranging from 0.9 to 58.5% for the Nucleocapsid protein and S1, S2, receptor binding domain, and ectodomain of the Spike protein^2–11^. Many estimates based on serological surveillance provide higher estimates of exposure than early estimates derived from case counts and case-based surveillance. Numerous hypotheses have been proposed to explain this higher-than-expected seropositivity in Africa: lack of testing coverage in symptomatic cases, high potential prevalence of asymptomatic and paucisymptomatic cases, cross-reactivity due to other circulating coronaviruses, or ELISAs that have high rates of false positives in sub-Saharan African populations^2,3,8,9,12–14^. This cross-reactivity has been associated with *Plasmodium* parasite load (though differences in acute malaria infection were not statistically significant)^12^ and IgG directed against malarial antigens, measuring past malaria exposure^14^. More generally, false-positives have been described for SARS-CoV-2 antibody testing due to various endogenous and exogenous factors^15^.

In this study, we investigate whether the cross-reactivity to S1 subunit of the Spike protein is associated with malaria exposure, and we investigate the relationship with *Plasmodium* species, malaria infection status, and age. We further examine whether cross-reactivity is associated with exposure to other human coronaviruses. Since no studies to date have determined the target of cross-reactive antibodies, we seek to define the molecular target, identify methods to reduce cross-reactivity, and determine whether cross-reactive antibodies could functionally protect subjects through neutralization.

## Materials and methods

### Summary

We used ELISAs to test whether pre-pandemic samples from people with and without malaria showed antibody responses to the S1 subunit of the SARS-CoV-2 Spike protein. We then conducted ELISAs using deglycosylated S1 Spike proteins to determine if cross-reactivity was affected by the glycosylation of the S1 protein. Next, we compared the results of our SARS-CoV-2 S1 Spike ELISAs to results of other SARS-CoV-2 ELISAs using the receptor binding domain of Spike, the Nucleocapsid protein, and a combined S2 Spike and Nucleocapsid.We also tested whether cross-reactivity can be reduced with the Microimmune SARS-COV-2 Hybrid Double Antigen Bridging Assay (DABA)^16^ or with an ELISA protocol modification incorporating an avidity wash with urea^14^. To test whether pre-pandemic samples reactive to S1 Spike correlated to immune responses to other antigens, we tested sample reactivity to a very large number of peptide and protein arrays using Rapid Extracellular Antigen Profiling and Serimmune. Lastly, we conducted viral neutralization assays to determine if reactive pre-pandemic samples were able to neutralize SARS-CoV-2 by blocking viral entry.

### S1 subunit ELISA

Serum (diluted 1:50) was used for all cohorts except CAM, for which dried blood spots were used. Samples were diluted to a final concentration equivalent to 1:50 serum dilution in dilution buffer (phosphate buffered saline, 0.1% Tween20, and 1% milk powder). S1 subunit Spike (Acro Biosystems; S1N-C52H2) enzyme-linked immunosorbent assay (ELISA) was performed as described previously^17^, except samples was not treated with Triton X-100 and RNase A, and plates were incubated for 2 h after adding blocking solution. Positive controls for IgG (camelid monoclonal nanobody VHH72-Fc antibody, reactive against SARS-CoV-2 Spike protein^18^, 34 ng/ml, in duplicate), and IgM (pooled convalescent serum diluted 1:100, in duplicate) and negative controls (healthy patient serum, from Sigma-Aldrich, in quadruplicate) were included. The optical density (OD) of a sample was calculated as the 450 nm value minus the 570 nm value. Sample OD was normalized by dividing the result by the mean of the respective IgG or IgM positive controls. Positivity was defined as normalized IgG OD above 0.1557969 and normalized IgM OD above 0.2810049 (mean + 3 standard deviations of the HCW neg cohort, which consisted of 80 healthy RT-qPCR negative US healthcare worker [HCW] controls).

### Deglycosylation ELISAs

ELISAs with the Spike S1 subunit treated to different conditions were performed in the same way as the native S1 subunit ELISA, except that the S1 protein was first altered according to each condition. S1 native was the unaltered S1 protein. Neuraminidase treatment: For S1 treated with neuraminidase to remove sialic acid, 2.8 $\upmu$g native S1 Spike protein was brought up to 20 $\upmu$l in water and 2 $\upmu$l neuraminidase stock (Sigma-Aldrich, N7885-2UN) was added and incubated at 37 $^\circ$C for 1 h. PNGase F treatment: For S1 treated with PNGase F to remove N-linked glycans, 20 $\upmu$g of S1 native was added to 40 $\upmu$l water. Then, 4 $\upmu$l 10x Glycoprotein Denaturing Buffer (New England Biosystems) was added, and the mixture was heated to 100 $^\circ$C for 10 min. To this mixture, 8 $\upmu$l of 10X GlycoBuffer 2 (New England Biosystems), 8 $\upmu$l of 10% NP-40 (New England Biosystems), 4 $\upmu$l PNGase F (New England Biosystems), and 16 $\upmu$l water were added. This mixture was then incubated for 1 h at 37 $^\circ$C. Denaturing: For denaturating conditions, 20 $\upmu$g of S1 native was added to 40 $\upmu$l water. Then, 4 $\upmu$l 10x Glycoprotein Denaturing Buffer (New England Biosystems) was added, and the mixture was heated to 100 $^\circ$C for 10 min.

All proteins treatment conditions were adjusted to a final concentration of 2 $\upmu$g/ml in PBS, the standard coating concentration for the ELISA. Deglycosylation ELISAs were run with three positive controls: pooled convalescent serum diluted 1:100; camelid monoclonal nanobody VHH72-Fc antibody, 34 ng/ml; and Anti-SARS-CoV-2 Spike RBD Antibody, Chimeric mAb, Human IgG1 (S1N-M122) (Acro Biosystems), 8 ng/ml. Serum from healthy people (Sigma-Aldrich) was used as a negative control. Percent of S1 native OD for sample *i* with protein treatment P was calculated by: (OD$_{i,P}$-OD$_{Neg,P}$)/(OD$_{i,S1} -$ OD$_{Neg,S1}) \times 100\%$, where OD = IgG OD value, *i* = sample, P = protein treatment, Neg = negative control, and S1 = native S1.

### ELISAs for receptor binding domain of Spike and Nucleocapsid

74 samples acutely infected with *P. falciparum* (confirmed with rapid diagnostic test and microscopy) from the SEN2 cohort were tested by ELISA for ectodomain of Spike and Nucleocapsid, as previously described using proteins expressed in baculovirus cells^19,20^. Continuous results of the S1 Spike protein ELISA were compared with continuous results of Spike ectodomain ELISA and Nucleocapsid protein ELISA using Pearson correlations, and binary results using cutoffs were compared using Chi-squared tests with Yates’s corrections.

### ELISAs for combined Spike S2 and Nucleocapsid

120 samples from the SEN1 cohort (acutely infected *P. falciparum* positive and negative timepoints) were tested with a commercial test including S2 subunit and Nucleocapsid combined (Omega Diagnostics) using proteins expressed in HEK293 cells.

### Rapid extracellular antigen profiling (REAP)

Rapid extracellular antigen profiling (REAP) was performed as previously described^17^ on 131 samples from 21 subjects from the SEN2 and BUR1 cohorts. This high-throughput discovery method enables detection of antibody reactivities against 2770 human extracellular folded proteins displayed on the surface of yeast. Briefly, serum from subjects is displayed against this library, IgG coated cells are magnetically isolated, and antigen identity is determined through sequencing. The “REAP Score” is determined from each antibody:antigen binding event, based on the enrichment of each protein’s barcodes before and after selection, with a score of 1.5 used as a cutoff for positivity. The RBD of the following coronaviruses were included in the library, as has been done previously: SARS-CoV-1, SARS-CoV-2, MERS-CoV, HCoV-OC43, HCoV-NL63, and HCoV-229E^17^.

### Serimmune peptide array for antibody binding specificity

For identification of antibody binding specificities in 74 samples from 69 subjects from Thiès, Senegal (SEN2), the Serum Epitope Repertoire Analysis (SERA) assay used a fully random 12-*mer* peptide library displayed by bacteria as described previously^21,22^. *Escherichia coli* was grown to express a library of 8 $\times$ 10$^{10}$ peptides and antibodies in serum bound to expressed antigen mimic peptides. The Protein-based Immunome Wide Association Study (PIWAS) algorithm was used to determine associations between immune profiles and exposure to disease, as has been done in previous studies^22^. Peptide motifs representing epitopes or mimotopes of malaria-specific antibodies were discovered using the IMUNE algorithm as previously described^23^. IMUNE compared IgG antibody repertoires from subjects with malaria who tested positive on SARS-CoV-2 Spike S1 ELISA (n = 23) with subjects with malaria who tested negative for Spike S1 ELISA (n = 28).

### Alternative assays and methods to reduce cross-reactivity

A urea wash to disrupt low avidity bonds was added to the S1 subunit ELISA, as previously described^14^. Urea is a chaotropic agent that can disrupt non-covalent bonds such as hydrophobic interactions, hydrogen bonds, and van der Waals forces^24–26^. When included in a modified ELISA as an avidity assay (AI), chaotropic agents can disrupt weak and low-avidity interactions between antibodies with their target antigen such that only high avidity antibodies will remain. In this study, we employ a modified ELISA as our avidity assay, similar to what has been used by others^14,24^, to remove low-avidity antibodies. After samples were added and incubated for 2 h, plates were washed 3 times, and 100 $\upmu$l of 2 M, 4 M, or 8 M urea in PBS was added to each well and plates were incubated at room temperature for 10 min before continuing with the protocol. A final 4 M Urea concentration was selected for further experiments (Supplemental Fig. S1). Positivity was defined as normalized IgG OD above 0.233 (mean + 3 standard deviations of IgG OD of 80 healthy RT-qPCR negative US healthcare worker (HCW) controls) tested by the same assay with the urea wash. Percent of S1 native OD for sample *i* with treatment P was calculated by: (OD$_{i,P}-$ OD$_{Neg,P}$)/(OD$_{i,S1} -$ OD$_{Neg,S1}) \times 100\%$, where OD = IgG OD value, *i* = sample, P = treatment, Neg = negative control, and S1 = native S1.

The Hybrid DABA measures reactivity to S1 and RBD antigens in a two-step double antigen binding format to reduce non-specific binding and was performed according to manufacturer instructions^16^. Cutoffs were calculated from the OD values of 3 negative controls included with the kit, with an upper cutoff for positivity, and a lower cutoff for negativity, and results falling between the two considered between which results were equivocal. The S1 subunit ELISA was also performed with IgG subclass-specific secondary antibodies to determine IgG subclasses IgG1, IgG2, IgG3, and IgG4 (Southern Biotech).

### Viral neutralization assays: pseudotyped virus (VSV-Spike-RLuc)

20 SEN2 cohort samples with a range of S1 Spike IgG values were tested to determine if sera was neutralizing against pseudotyped virus. HEK293T cells (ATCC) were used to produce Vesicular stomatitis virus (VSV)-based pseudovirus particles^27,28^. Cells were transfected via calcium phosphate method with a pCAGGS vector plasmid expressing the Wuhan-Hu-1 spike glycoprotein (NR-52310, produced under HHSN272201400008C and obtained through BEI Resources, NIAID, NIH) and were then inoculated with a replication-deficient VSV encoding Renilla luciferase (Rluc) instead of the VSV-G glycoprotein open reading frame. HEK293T cells were cultured in DMEM (Gibco) supplemented with 5% Fetal Bovine Serum (VWR) and 1% Penicillin/Streptomycin (Gibco) at 37 $^\circ$C/5% CO$_2$. After 1 h, the inoculum was removed and cells were washed with PBS. Media containing anti-VSV-G clone IE9F9 was then added to neutralize any residual VSV-G. Pseudovirus was harvested 24 h later and cellular debris was removed by centrifugation at 3000 rpm for 10 min. Virus was stored at $-80\, ^\circ$C until experiments were performed.

Vero-E6 cells were obtained from ATCC and cultured in DMEM (Gibco) supplemented with 5% Fetal Bovine Serum (VWR) and 1% Penicillin/Streptomycin (Gibco) at 37 $^\circ$C/5% CO$_2$. $1\times 10^4$ Vero-E6 cells in 100 $\upmu$l total volume were seeded in black-walled clear bottom 384-well plates (Corning) and incubated overnight at 37 $^\circ$C. The next day, patient sera dilutions were prepared in concentrations of 1:10–1:1280 and were pre-incubated with Spike-expressing VSV pseudovirus for 1 h at room temperature with gentle agitation. Pseudovirus-sera mixtures were added to the adhered Vero-E6 cells at a final virus concentration of 1:10 volume/volume and incubated at 37 $^\circ$C for 24 h. Cells were lysed using the Renilla Luciferase Assay System (Promega) according to manufacturer instructions. Luciferase activity was measured by relative light units using a microplate reader (BioTek Synergy or Cytation 5). Percent entry after neutralization was calculated by dividing luminescence at a given serum dilution to that of a no serum control. Means of samples tested in duplicate were used. One replicate for a positive control failed, thus only one data point is provided in this run. Percent entry among samples was compared against percent entry of a positive neutralization control (serum from a COVID-19 positive inpatient) using a one-sample t-test.

### Statistical analysis

Cohorts with 3 or fewer samples were excluded from analysis. Statistics were performed in RStudio (Version 1.2.5001). Comparisons for two continuous variables were performed with Welch’s unequal variances t-tests. Comparisons for binary variables were performed with Chi-squared test with Yates’s correction or Fisher’s exact tests depending on the sample size. To compare more than 2 continuous variables, one-way ANOVAs were used, and when significant, were followed by Tukey’s HSD. Normality was checked using Q–Q plots and natural log transformations were used where appropriate to ensure data was normally distributed. Reported p values are Bonferroni corrected for the number of tests performed. Continuous variables were transformed into binary variables using cutoffs for positivity calculated from normalized IgG and IgM values from 80 RT-qPCR negative healthcare workers (mean + 3 SDs). To identify effects of age quartile (< 9 years, 9 to < 18 years, 18 to < 32.5 years, 32.5 years and up) and malaria status (subjects with acute infection, uninfected subjects in malaria endemic areas, and uninfected subjects in non-endemic areas), log-transformed multivariate linear regression models were performed using normalized IgG and IgM (separately) as dependent variables and age quartile and malaria status with interaction terms as independent variables. For subjects with missing age data, subjects were categorized into age quartile based on the cohort’s inclusion criteria when possible. Boxplots show minimum, 1st quartile, median, 3rd quartile and maximum (excluding outliers).

### Ethical approval

CAM (Cameroon): Ethical approval for this study was granted by the University of Doula Institutional Ethics Committee for Research on Human Health (Protocol No 1617), the Institutional Review Board of the Doula Military Hospital (IRB 0180776), and the Human Investigation Committee of Yale University (Protocol 2000023509). All samples were collected with informed consent and in accordance with all ethical requirements of the Institutional Review Boards of the University of Doula, Doula Military Hospital, and Yale University.

SEN1 (Kédougou, Senegal): This study was conducted with ethical approval from the National Ethics Committee of Senegal (CNERS) and the Institutional Review Board of the Yale School of Public Health. All research was performed in accordance with relevant guidelines and regulations, and informed consent was obtained from all participants and/or their legal guardians. Samples used in this study were collected as part of ongoing surveillance conducted by Institut Pasteur de Dakar investigating causes of febrile illness.

SEN2 (Thiès, Senegal): Ethical approval for this study was granted by the National Ethics Committee of the Ministry of Health in Senegal (Protocol SEN 14/49), the Institutional Review Board of the Harvard T.H. Chan School of Public Health (IRB 14-2830), and the Human Investigation Committee of Yale University (Protocol 2000023287). All samples were collected with informed consent and in accordance with all ethical requirements of the National Ethics Committee of Senegal, Institutional Review Board of the Harvard T.H. Chan School of Public Health, and the Human Investigation Committee of Yale University.

BUR1 and BUR2 (Burkina Faso): Ethical approval for these studies was granted by the Institutional Review Boards of Centre Muraz (Ref. A003-2013/CE-CM), the Centre National de la Recherche Scientifique et Technologique (Protocols Ref. A14-2016/CEIRES; A016-2017/CEIRES) and the Human Investigation Committee of Yale University (Protocols 1209010884 and 2000021308). All samples were collected with informed consent and in accordance with all ethical requirements of the Institutional Review Boards of the Centre Muraz, CEIRES, and Yale University.

GHA (Ghana): These studies were approved by the Yale University Human Investigations Committee and the Institutional Review Boards at the Noguchi Memorial Institute for Medical Research, the Ghana Health Service, and the Scientific Review Committee and the Institutional Ethics Committee at the Kintampo Health Research Center. In addition, District Ministry of Health representatives and District Ministry of Education representatives approved the study and assisted in communication with participating schools.

COL1 and COL2 (Colombia): This study was approved by the Committee of Ethics in Research of the Facultad Nacional de Salud Pública (meeting of 22 May 2014) of the Universidad de Antioquia. All the study participants gave informed consent.

BRA (Brazil): Ethical approval for was granted by the Human Investigation Committee of Yale University (HIC# 1006006956) and from the CONEP in Brazil (CONEP/Brazil 45217415.4.0000.0040).

NEP (Nepal): Ethical approval for this study was granted by the Nepal Health Research Council (Reg 106/2013) and the Stanford University Institutional Review Board (IRB 29992). All samples were collected with informed consent and in accordance with all ethical requirements of the Ethical Review Board of the Nepal Health Research Council and the Institutional Review Board of Stanford University.

EBV (USA): This study was approved by all relevant the Institutional Review Boards (i.e., those of Northwestern University, the Ann & Robert H Lurie Children’s Hospital of Chicago and DePaul University).

HCW and COVID-19 (USA): This study was approved by the Yale Human Investigation Committee, protocol #2000027690. All samples were collected with informed consent and in accordance with Yale IRB approval.

YNHH (USA): A de-identified *P. vivax* infected blood sample was obtained under an approval protocol to Choukri Ben Mamoun.

## Results

### High degree of cross-reactive antibody responses identified in malaria-infected and exposed individuals

**Figure 1:**
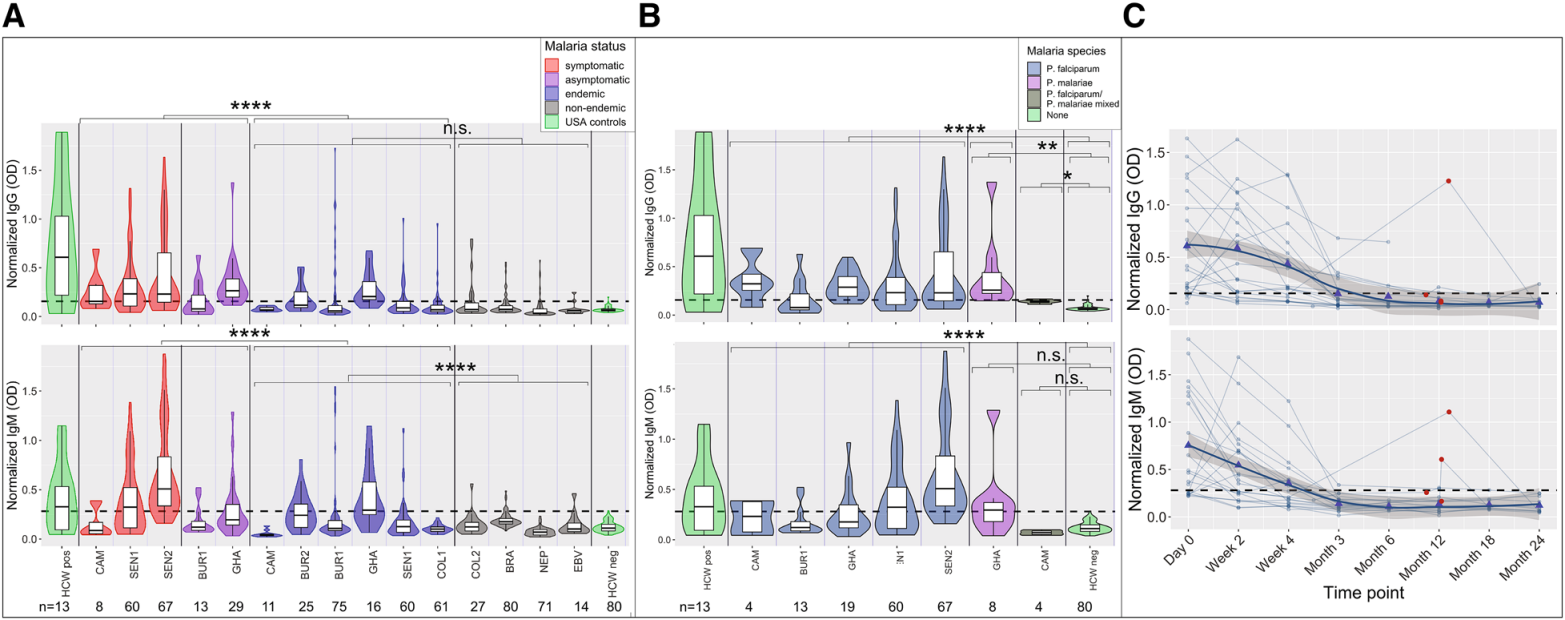
High frequency of cross-reactive antibodies to SARS-CoV-2 Spike protein from *Plasmodium*-infected individuals. In (**A**,**B**), Violin plots showing normalized IgG and IgM responses among subjects from different cohorts. ****p < 0.0001, ***p < 0.001, **p < 0.01, *p < 0.05, *n.s*. not significant. (**B**) Subjects with acute malaria infection (symptomatic and asymptomatic) had significantly higher IgG and IgM than uninfected subjects living in malaria endemic areas (t-test p value < 0.0001 for both log IgG and log IgM). IgM but not IgG cross-reactivity was also significantly higher among uninfected individuals living in malaria endemic settings with previous exposure compared to individuals in non-endemic settings (t-test log IgG p value = 0.367 and log IgM p value < 0.0001). (**B**) Of malaria positive subjects, 163 had *P. falciparum* mono-infection (107 IgG positive and 98 IgM positive), 8 had *P. malariae* mono-infection (6 IgG positive and 4 IgM positive), 6 had *P. falciparum/P. malariae* mixed infections (3 IgG positive and 0 IgM positive), and 1 had *P. vivax* mono-infection (0 IgG or IgM positive). Log normalized IgG was significantly higher among subjects with *P. falciparum*, *P. malariae*, and mixed infections than among negative controls (t-test p value < 0.0001 for log *P. falciparum*, p value = 0.002 for *P. malariae* and p value = 0.013 for mixed infections), and normalized IgM was significantly higher among subjects with *P. falciparum* but not *P. malariae* or mixed infections than among negative controls (t-test p value < 0.0001 for *P. falciparum*, p value = 0.222 for *P. malariae* and p value = 1 for mixed infections). Normalized IgG and IgM was not significantly different between subjects with *P. falciparum* and *P. malariae* (t-test p value = 1 for IgG and p value = 1 for IgM). (**C**) Normalized IgG and IgM over time in 21 subjects with *P. falciparum* mono-infection on Day 0. Both IgG and IgM peaked between Day 0 and Week 4 for all subjects. Reinfection, confirmed by rapid diagnostic test and microscopy and shown by red circles, boosted IgG response in 1 of 4 subjects and IgM response in 2 of 4 subjects. Bold trend line based on local regression (LOESS). In (**A**–**C**), normalized IgG or IgM calculated by IgG or IgM OD divided by IgG or IgM of positive control (camelid monoclonal chimeric nanobody VHH72 antibody was IgG control, and pooled SARS-CoV-2 convalescent serum was IgM control). Samples were run in singlicate, duplicate or triplicate as sample volume allowed. Black dashed lines represent cutoffs for positivity, calculated from normalized IgG and IgM values from 80 RT-qPCR negative healthcare workers (HCWs) (mean + 3 SDs).

**Figure 2:**
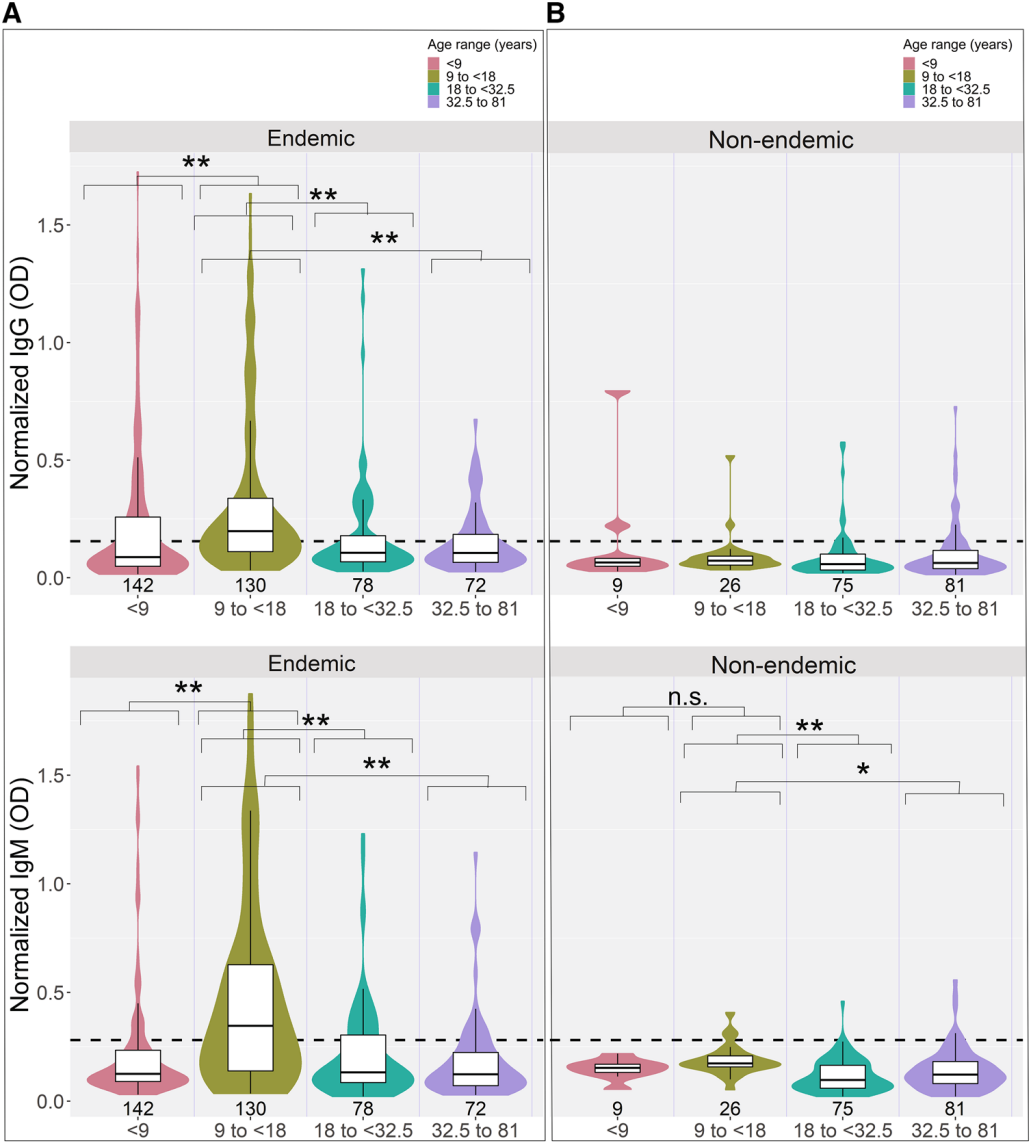
S1 subunit Spike cross-reactivity differs by age. Violin plots showing normalized IgG and IgM responses among subjects living in (**A**) endemic countries (including those with and without acute malaria infection) and (**B**) non-endemic countries. Age is divided by quartiles representing under 9 years, 9–18 years, 18–32.5 years, and 32.5 years and older. Top plots show normalized IgG and bottom plots show normalized IgM. Normalized IgG or IgM calculated by IgG or IgM OD divided by IgG or IgM of positive control (camelid monoclonal chimeric nanobody VHH72 antibody was IgG control, and pooled SARS-CoV-2 convalescent serum was IgM control). Black dashed lines represent cutoffs for positivity, calculated from normalized IgG and IgM values from 80 RT-qPCR negative HCWs (mean + 3 SDs). ****p < 0.0001, ***p < 0.001, **p < 0.01, *p < 0.05, *n.s*. not significant.

We identified a high degree of cross-reactivity to SARS-CoV-2 Spike protein (S1 subunit) in individuals with acute (symptomatic and asymptomatic) malaria infection (Fig. 1A,B) by ELISA. A total of 741 samples from 617 individuals from 8 countries (Table 1, Table S1) collected before the first reported case of COVID-19 were tested for Spike protein seropositivity. The observed cross-reactivity was significantly higher in individuals with acute *Plasmodium* infection compared to uninfected individuals in malaria endemic areas (t-test log IgG and IgM p values < 0.0001, Fig. 1A,B). Individuals with symptomatic malaria infection had significantly higher cross-reactive log IgM but not log IgG than asymptomatic individuals (t-test log IgG p value = 1 and log IgM p value = 0.0001). IgM but not IgG cross-reactivity was also significantly higher among uninfected individuals living in malaria endemic settings with previous exposure compared to individuals in non-endemic settings (t-test log IgG p value = 0.367 and log IgM p value <0.0001). These patterns are similar for people across age quartiles (Fig. 2). When split by age quartile, one-way ANOVAs for log normalized IgG and IgM among people in endemic areas were significant (both p values < 0.0001). In endemic areas, age group 9 to < 18 years had significantly higher log normalized IgG and IgM than the other 3 quartiles (p values < 0.001 for all three quartiles for both IgG and IgM). One-way ANOVA for log normalized IgG among people in non-endemic areas was not significant (p value = 0.47) but was significant for IgM (p value = 0.0004). IgM for 9 to < 18 year old age group was only significantly higher than 18 to < 32.5 year and 32.5–81 year age groups (p value < 0.001 and p value = 0.016, respectively).

In S1-reactive antibody responses in 131 longitudinal samples from 21 Senegalese subjects, IgG and IgM responses peaked 0–4 weeks post *P. falciparum* infection for all subjects, decreased with time, and, in some subjects, were boosted by subsequent reinfections (boosting in 1 of 4 IgG samples and 2 of 4 IgM samples with confirmed *P. falciparum* subsequent re-infection) (Fig. 1C).

**Figure 3:**
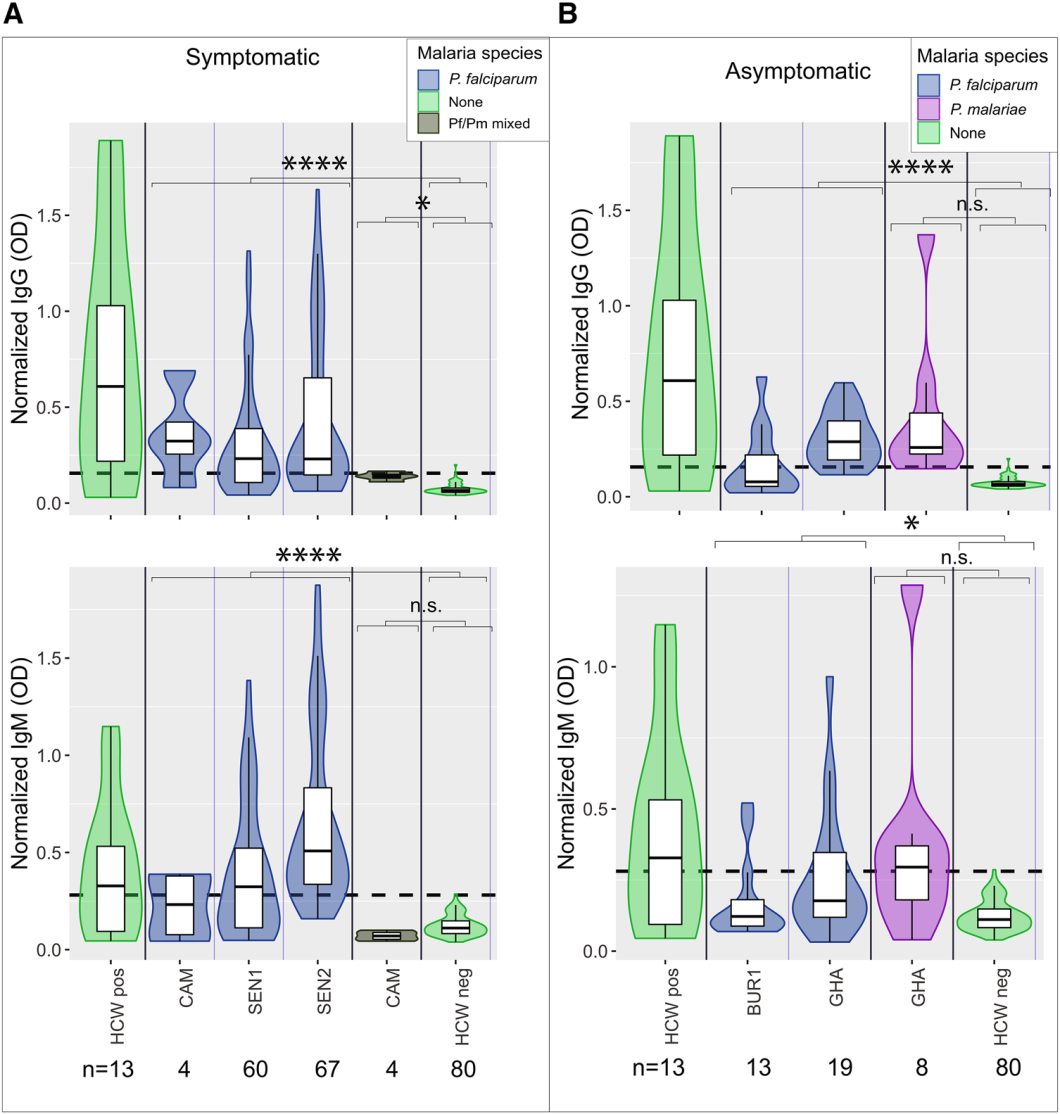
*Plasmodium falciparum* and *P. malariae* is associated with S1 subunit Spike cross-reactivity in symptomatic and asymptomatic subjects. Violin plots showing normalized IgG and IgM responses among (**a**) symptomatic and (**b**) asymptomatic subjects by species of malaria infection. For symptomatic subjects, both IgG and IgM were significantly higher among subjects with *P. falciparum* than healthy US HCW controls (unequal variances t-tests p values < 0.0001 for both IgG and IgM). Subjects with symptomatic *P. falciparum/P. malariae* mixed infections had significantly higher log IgG but not IgM than healthy US HCW controls (unequal variances t-tests p values = 0.0013 for IgG and p value = 0.222 for IgM). For asymptomatic subjects, both log IgG and IgM were significantly higher among subjects with *P. falciparum* than healthy US HCWs controls (unequal variances t-tests IgG p value < 0.0001 and IgM p value = 0.016). Asymptomatic subjects with *P. malariae* had significantly higher log IgG but not log IgM than healthy US HCWs controls (unequal variances t-tests IgG p values = 0.002 and IgM p value = 0.222). Normalized IgG or IgM was calculated by IgG or IgM OD divided by IgG or IgM of positive control (camelid monoclonal chimeric nanobody VHH72 antibody was IgG control, and pooled convalescent serum from SARS-CoV-2 patients was IgM control). Black dashed lines represent cutoffs for positivity, calculated from normalized IgG and IgM values from 80 RT-qPCR negative HCWs (mean + 3 SDs). ****p < 0.0001, ***p < 0.001, **p < 0.01, *p < 0.05, *n.s*. not significant.

Among malaria-positive subjects (acutely infected, combined symptomatic and asymptomatic), log normalized IgG was significantly higher among subjects with *P. falciparum*, *P. malariae*, and *P. falciparum/P. malariae* mixed infections than among unexposed controls (controls were healthy US HCWs; t-test p value < 0.0001 for log *P. falciparum*, p value = 0.002 for *P. malariae* and p value = 0.013 for mixed infections) (Fig. 1B). Log normalized IgM was significantly higher among subjects with *P. falciparum* than log IgM in unexposed controls (t-test p value < 0.0001). Log normalized IgM was not significantly different among subjects with *P. malariae* or mixed infections than among unexposed controls (p value = 0.222 for *P. malariae* and p value = 1 for mixed infections). However, the latter comparison was limited by few subjects with non-*P. falciparum* malaria. Patterns were similar when symptomatic and asymptomatic subjects were considered separately (Fig. 3). Thus, our results suggest that both *P. falciparum* and *P. malariae* are capable of inducing cross-reactive IgG, and in most cases, IgM antibodies.

Since *P. falciparum* infection can induce polyreactive B cells^29^, we investigated this potential explanation for SARS-CoV-2 cross-reactivity. Sera from patients with Epstein Barr Virus (EBV), a disease that, like *P. falciparum*, can induce characteristic polyreactive B cell responses, was found to have significantly less reactivity than sera from subjects with acute malaria infection (t-test p values < 0.0001 for both IgG and IgM when compared to two separate time points after EBV infection, generally 6 weeks and 6 months after infection), indicating that cross-reactivity is not necessarily correlated with broad, disease-independent poly reactive B cell responses (Fig. 1A).

**Table 1:** Seropositivity rates of cohorts.

Cohort	N	IgG positive N (%)	IgM positive N (%)
HCW pos	13	10 (77%)	8 (62%)
CAM	19	4 (21%)	2 (11%)
SEN1	120	53 (44%)	40 (33%)
SEN2	67	45 (67%)	56 (83%)
BUR1	88	21 (24%)	10 (11%)
GHA	45	37 (82%)	21 (47%)
BUR2	25	10 (40%)	12 (48%)
COL1	61	11 (18%)	0 (0%)
COL2	27	6 (22%)	1 (4%)
BRA	80	14 (18%)	9 (11%)
NEP	71	6 (8%)	0 (0%)
EBV	14	2 (14%)	1 (7%)
HCW neg	80	1 (1%)	1 (1%)

### Lack of correlated cross-reactivity between anti-S1 antibodies and other human coronavirus proteins

In 74 samples from the SEN2 cohort, we observed limited correlated cross-reactivity between Spike S1 IgG and other SARS-CoV-2 proteins (Spike ectodomain: Pearson’s R = 0.062, p value = 0.60, and Nucleocapsid: Pearson’s R = 0.17, p value = 0.15). In 120 samples from the SEN1 cohort, we also did not find that S1 Spike cross-reactivity was significantly correlated with results of a commercial SARS-CoV-2 antibody that measured combined reactivity to Spike S2 subunit and Nucleocapsid proteins (Chi-squared p value = 0.319).

**Figure 4:**
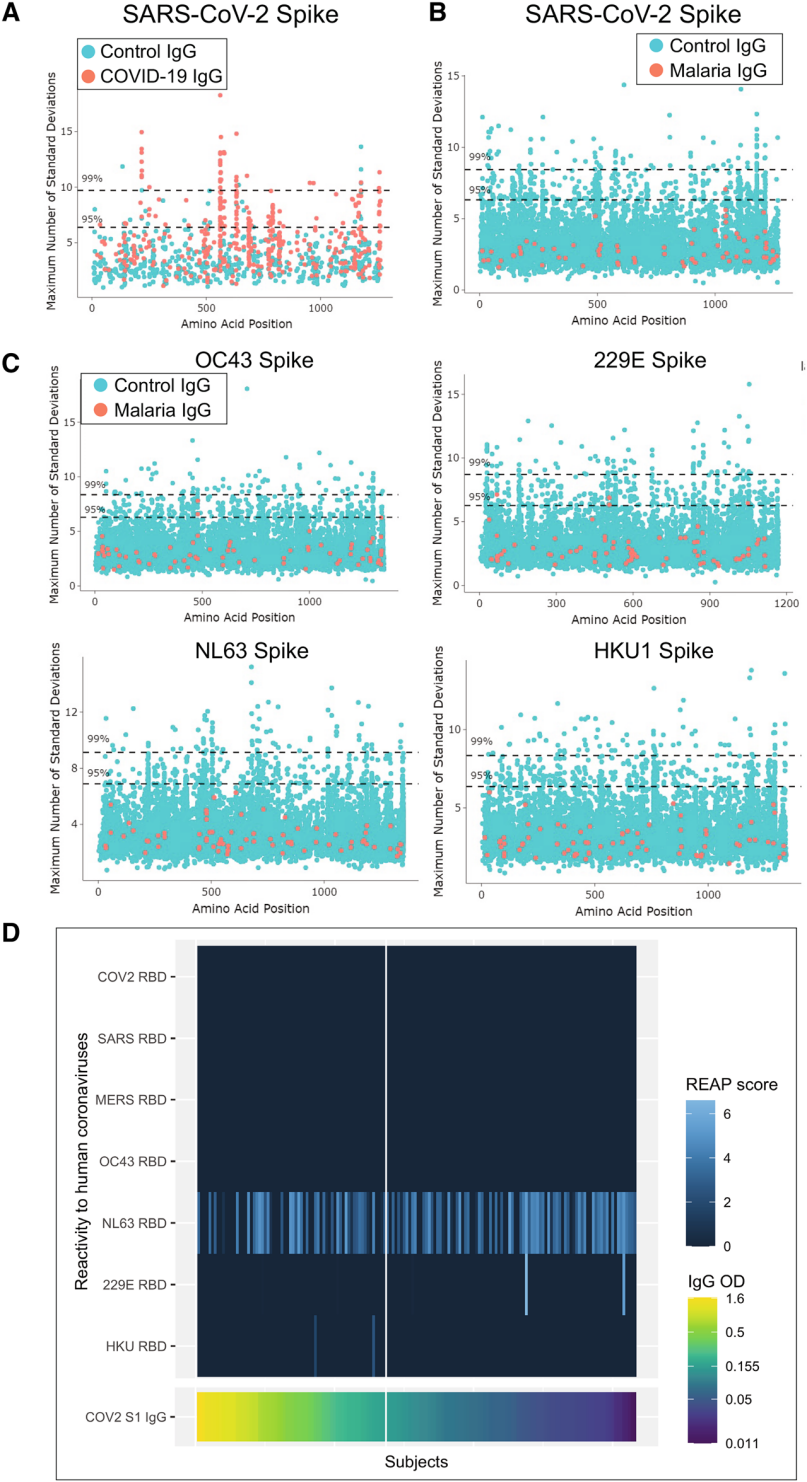
Cross-reactive IgG is not associated with reactivity to SARS-CoV-2 or other coronavirus peptides or RBD proteins. PIWAS plots displaying the maximum *k*-mer enrichment for each subject across the entire SARS-CoV-2 Spike protein, with conserved peaks representing reactivity to conserved epitopes. (**A**) Positive control demonstrating IgG COVID-19 cases (red) and COVID-negative controls (blue). (**B**,**C**) PIWAS *k*-mer enrichments for IgG from acute malaria cases (shown in red in **B**,**C**) on the four common coronavirus proteomes compared to 8243 pre-pandemic controls (shown in blue). The Y-axis represents the standard deviation calculated from the control values. The maximum *k*-mer peak for each individual is shown. Dashed lines represent significance levels of 95% and 99%. (**D**) Cross-reactivity to other human coronaviruses assessed by yeast expressed exported protein array (REAP) is only observed for NL63 and did not differ between S1 positive and S1 negative subjects (S1 positives on the left side of the vertical white line and S1 negatives on the right side). REAP score cut-off of 1.5 is considered a positive response. IgG to S1 from subjects are ordered by descending normalized OD (shown in bottom plot).

To test whether the cross-reactivity in malaria endemic regions was related to exposure to other alpha and beta-human coronaviruses, we used bacterially expressed peptide arrays^21–23^ and yeast expressed protein arrays known as Rapid Exoproteome Antigen Profiling (REAP)^17^ to test reactivity to the receptor binding domain (RBD) of human coronaviruses. Linear peptide epitopes tested against bacterial display libraries did not identify substantial binding of malaria-induced antibodies against SARS-CoV-2 peptides (Fig. 4B) or other coronavirus peptides (Fig. 4C). IgG from COVID-19 cases did show substantial enrichment against SARS-CoV-2 Spike compared to negative controls (Fig. 4A). Malaria peptide motifs were identified (Poisson p < 0.01), but no peptides to SARS-CoV-2 or other human coronaviruses were significantly enriched among samples that tested positive in the SARS-CoV-2 S1 Spike ELISA compared to samples that tested negative. Although previous studies suggested cross-reactivity to SARS-CoV-2 is caused by prior exposure to NL63, 229E^30^ and OC43^31^, we did not observe cross-reactivity between S1 positives by ELISA and peptides, SARS-CoV-2 RBD, or SARS-CoV-2 proteome using either approach except NL63, which showed overall high reactivity with REAP (Fig. 4D). While samples positive for NL63 (using the REAP cutoff of 1.5) and normalized IgG positives were not significantly related (Chi-squared test p value = 0.257), when the variables were binary, they were significantly negatively correlated (Spearman’s rank correlation coefficient = − 0.24, p value = 0.003). No samples tested with REAP showed binding to SARS-CoV-2 Spike RBD.

### The target of malaria-associated anti-S1 antibodies is sialic acid on N-linked glycans and does not neutralize SARS-CoV-2

**Figure 5:**
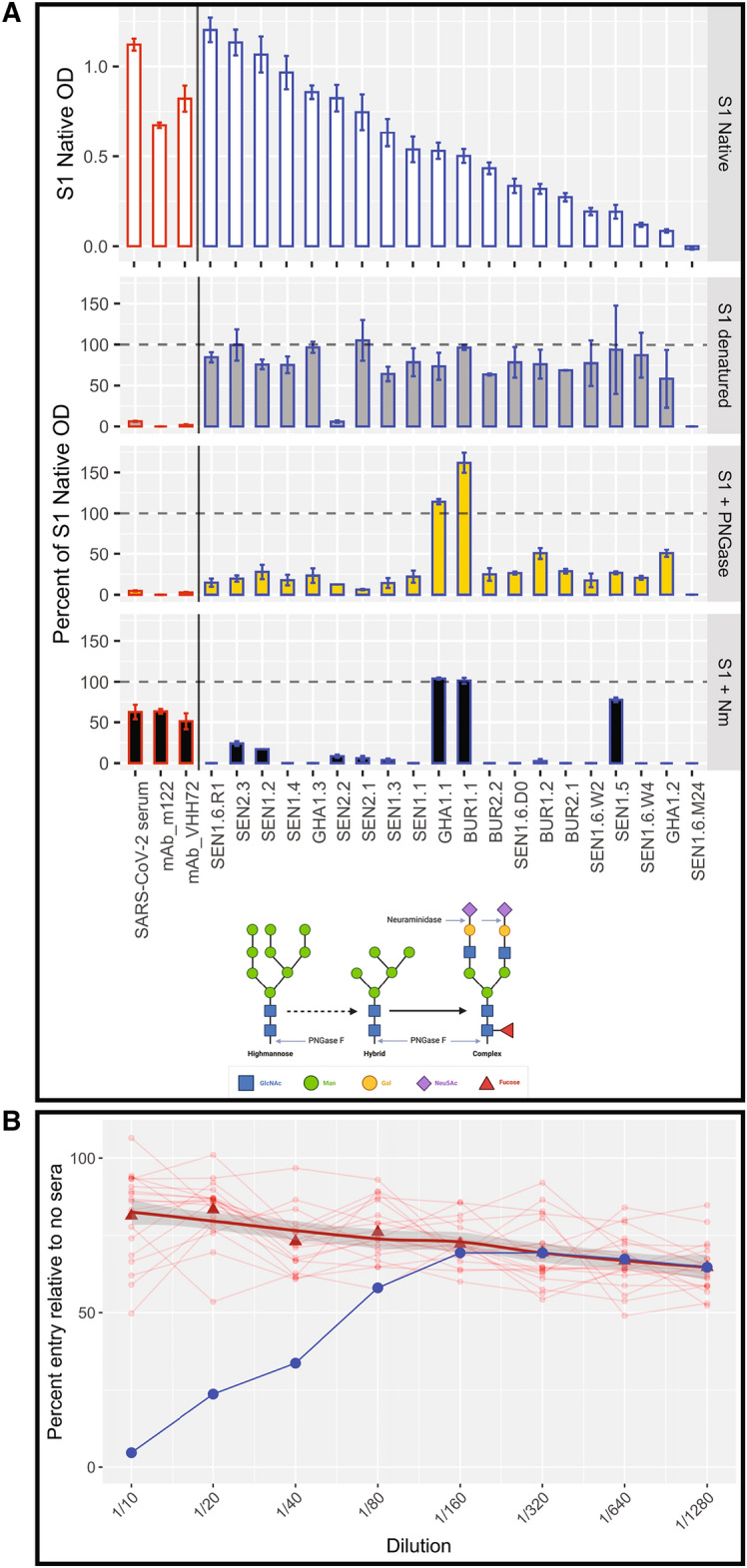
Cross-reactive IgG targets sialic acid on N-linked glycans and is not neutralizing. (**A**) S1 protein was subjected to three conditions to modify the structure: denaturing; treating with PNGase F to remove all N-linked glycans; and treating with neuraminidase to remove sialic acid. ELISA was performed on samples with each protein condition, and the percent of S1 Native OD under each condition is shown for 3 controls (pooled convalescent serum from SARS-CoV-2 patients, mouse monoclonal SARS-CoV-2 Spike M122 antibody, and camelid monoclonal SARS-CoV-2 Spike chimeric nanobody VHH72 antibody, all outlined in red) and 20 subjects (shown individually and each outlined in black). Neuraminidase treatment decreased samples to 23.0% (95% CI 1.1, 44.9) of OD of S1 native, significantly more than the reduction of SARS-CoV-2 convalescent serum (decreased to 63% of OD of S1 native), suggesting cross-reactivity was due to reactions with terminal sialic from glycosylated sites. Subjects are ordered by descending normalized OD IgG (shown in top plot). (**B**) In a neutralizing assay using pseudotyped viruses (VSV-Renilla luciferase pseudotyped with Spike), 20 samples (red circles) with high S1 Spike IgG ELISA reactivity showed no neutralization. Bold trend line based on local regression (LOESS) of samples. Control (serum from a COVID-19 positive inpatient, in blue) shows neutralization at dilutions of 1/40 and less.

To test the specificity of the cross-reactivity to either structural epitopes or glycosylation, we treated the S1 protein to alter its structure and composition of glycans and carbohydrates. In ELISAs, IgG cross-reactivity was maximally reversed upon treatment with neuraminidase, which removes the terminal sialic acids from glycosylated sites. Neuraminidase treatment caused a significantly greater reduction among cross-reactive malaria samples than in SARS-CoV-2 convalescent sera and SARS-CoV-2 Spike monoclonals, which were positive controls (Fig. 5A). Conversely, treatment with PNGase F (which removes all N-linked glycans) significantly reduced reactivity of cross-reactive antibodies, but also significantly reduced reactivity of controls. Denaturing the S1 protein demonstrated only modest decreases in reactivity for cross-reactive antibodies, however it eliminated binding for controls, implying a high degree of conformational epitopes. These results implicate cross-reacting antibody binding to terminal sialic acids of complex glycans in SARS-CoV-2 Spike S1. Of 22-N-linked glycosylation sites spanning the Spike protein, 52% are complex and fucosylated and 15% contain at least one sialic acid^32^. The majority of the N-linked glycans in the S1 domain of Spike protein (aa16-685) are complex, and are both fucosylated and sialated^32^. Three samples maintained high reactivity to S1 treated with neuraminidase (133.6%, 126.2%, and 131.2% of native S1 OD), suggesting that cross-reactivity in these samples could be caused by another mechanism.

We next sought to determine whether these malaria-induced antibodies could neutralize SARS-CoV-2 invasion, possibly providing protection. Cross-reactive antibodies did not demonstrate significant neutralizing activity via invasion at any dilution with pseudotyped virus (Fig. 5B) or SARS-CoV-2 virus (data not shown), corroborating a recent study^31^.

### Alternative assays increase specificity in malaria samples

**Figure 6:**
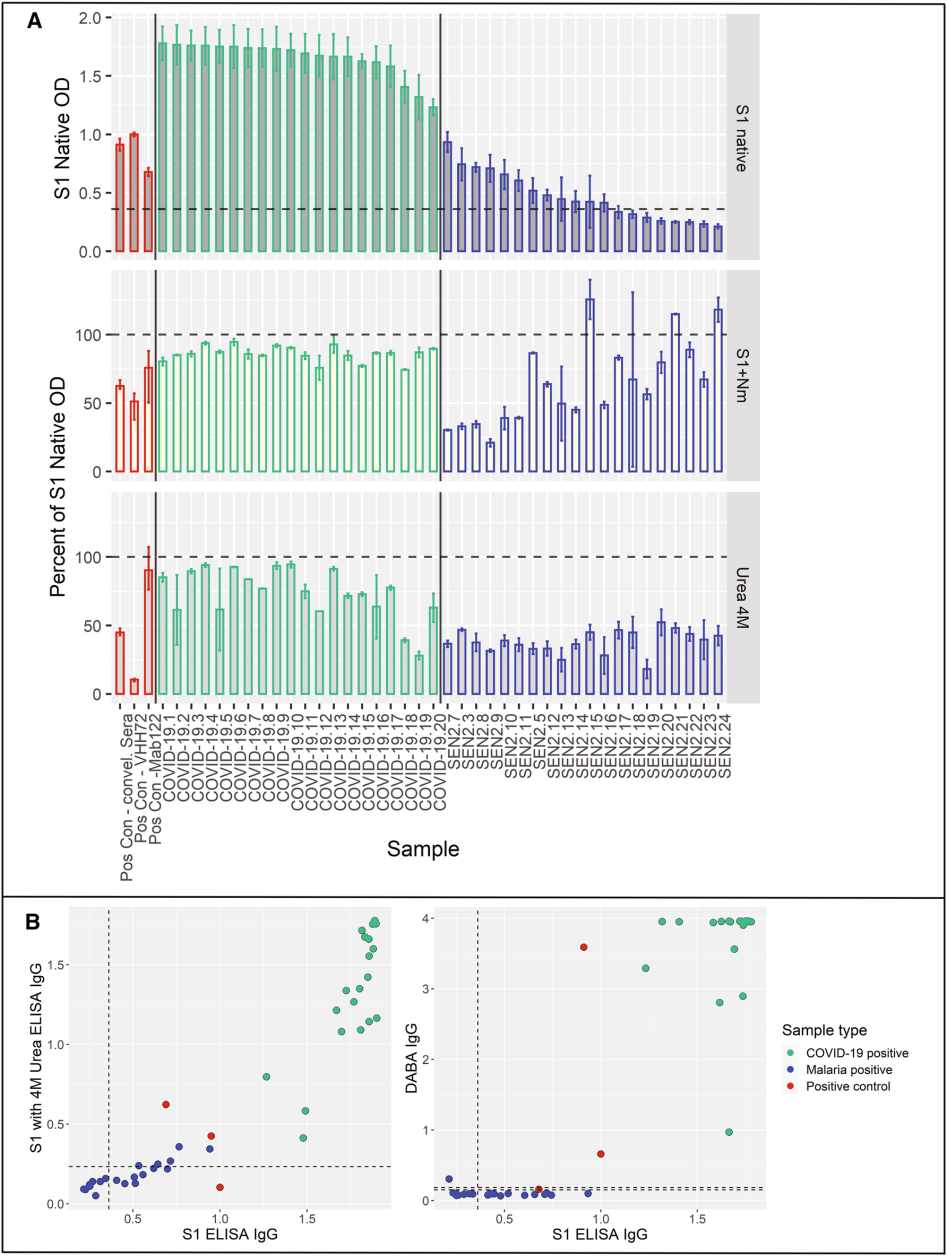
Neuraminidase, urea and Hybrid DABA reduce cross-reactivity in malaria samples. The S1 subunit ELISA was performed on 20 COVID-19 positive samples and 20 malaria positive samples in two alternative ways to increase its specificity. The S1 protein was first treated with neuraminidase to remove sialic acid and then the S1 subunit ELISA was performed with a wash of 4 M urea to reduce non-specific binding (performed in duplicate for samples and quadruplicate for controls, error bars show minimum and maximum values). The percent of S1 Native OD under each condition is shown for 3 controls (pooled convalescent serum from SARS-CoV-2 patients, mouse monoclonal SARS-CoV-2 Spike M122 antibody, and camelid monoclonal SARS-CoV-2 Spike chimeric nanobody VHH72 antibody, all outlined in red), 20 COVID-19 positive samples (shown individually and each outlined in green), and 20 malaria positive samples (shown individually and each outlined in blue). The urea wash decreased the IgG of malaria samples to 38.2% of S1 native, a significantly greater decrease than that of COVID-19 samples (73.8% of S1 native, t-test p value < 0.0001). Similarly, treating the S1 protein with neuraminidase reduced the IgG of malaria samples significantly more than reduction in COVID-19 samples (64.6% vs. 86.0% of S1 ELISA IgG values, t-test p value = 0.006). Subjects are ordered by descending normalized OD IgG (shown in top plot), with dashed line indicating cutoff for positivity. (**B**) Scatterplots showing values of S1 subunit ELISA vs. S1 subunit ELISA with 4 M urea wash and Hybrid DABA, two methods of increasing specificity for antibody reactivity. Three positive controls (pooled convalescent serum from SARS-CoV-2 patients, mouse monoclonal SARS-CoV-2 Spike M122 antibody, and camelid monoclonal SARS-CoV-2 Spike chimeric nanobody VHH72 antibody, in red), twenty COVID-19 positive samples (in green) and 20 malaria positive samples (in blue) were tested. For S1 ELISA, dashed lines represent cutoffs for positivity, and for DABA, lower and upper dashed lines represent cutoff for negativity and positivity, respectively (values between lines are equivocal). The 20 COVID-19 positive samples tested positive by both S1 subunit ELISA with and without urea wash. Of 20 malaria positive samples, 12 (60%) tested positive by S1 subunit ELISA, and 5 (25%) by S1 subunit ELISA with a 4 M urea wash (Fisher’s exact test p value = 0.243 for COVID-19 and malaria samples by S1 subunit ELISA with and without a urea wash). Twenty of 20 COVID-19 positive samples tested by Hybrid DABA tested positive, while only 1 (5%) of 20 malaria positive samples tested positive (12 of the 20 had tested positive by S1 subunit ELISA). The Hybrid DABA was significantly more effective at reducing cross-reactive positivity among malaria samples (Fisher’s exact test p value = 0.008).

Having identified that the molecular target of the cross-reactive malaria-associated antibodies was sialic acid, we hypothesized that these might represent low-avidity interactions that could be reduced with experimental modifications or alternatives. To this end we explored two alternative assays, an avidity assay with urea wash^14^ and a hybrid DABA assay^16^. Twenty COVID-19 inpatient samples all tested positive by S1 subunit ELISA and by S1 subunit ELISA with a 4 M urea wash (Fig. 6A,B). The 4 M urea concentration was selected to reduce false positivity after testing 2 M, 4 M, and 8 M urea concentrations on 28 malaria exposed samples (Figure S1). Of 20 samples from the SEN2 cohort (all with symptomatic *P. falciparum* malaria), 12 (60%) tested positive by S1 subunit ELISA, and 5 (25%) by S1 subunit ELISA with a 4 M urea wash (Fisher’s exact test p value = 0.243 for COVID-19 and malaria samples by S1 subunit ELISA with and without a urea wash) (Fig. 6B). When samples were tested by S1 subunit ELISA with a 4 M urea wash, COVID-19 inpatient samples had a mean IgG OD of 73.8% of S1 ELISA IgG values, and malaria samples had a mean IgG OD of 38.2% of S1 ELISA IgG values, making the avidity index for malaria samples (38.2%) significantly lower than that of COVID-19 positive samples (73.8%, t-test p value < 0.0001) (Fig. 6A). Similarly, treating the S1 protein with neuraminidase reduced the IgG of malaria samples significantly more than reduction in COVID-19 samples (64.6% vs. 86.0% of S1 ELISA IgG values, respectively, t-test p value = 0.006). As a percent of S1 native levels, the urea wash reduced reactivity significantly more than treating the S1 protein with neuraminidase for both COVID-19 positive and malaria positive samples (t-test p value = 0.0009 for COVID-19 positive samples and p value = 0.001 for malaria positive samples). When reactivity to IgG subclasses (IgG1, IgG2, IgG3, and IgG4) for the 20 COVID-19 inpatient samples and 20 malaria exposed samples were characterized, both COVID-19 samples and malaria samples were primarily reactive to IgG1 and IgG3 subclasses (Fig. S3).

Twenty of 20 COVID-19 positive samples tested by Hybrid DABA tested positive, while 1 (5%) of 20 malaria positive samples tested positive (12 of the 20 had tested positive by S1 subunit ELISA) (Fig. 6B). The Hybrid DABA was significantly more effective at reducing cross-reactive positivity among malaria samples (Fisher’s exact test p value = 0.008). Testing an additional 41 malaria samples (30 of which had been positive by the S1 ELISA), 2 samples were positive by DABA, 1 was equivocal, and 38 were negative (Figure S1).

## Discussion

Our study reveals that acute malaria infection can cause cross-reactivity to the S1 Spike protein through antibody binding to terminal sialic acids of complex glycans. Reactivity is more pronounced among people with diagnostically-confirmed acute malaria infection than those previously exposed to malaria but without acute infection. People with acute malaria infection could have stronger immune responses to malaria than people who were previously exposed but without acute infection, which could be related to our results that people with acute malaria infection had increased reactivity to Spike S1. Cross-reactivity did not neutralize by blocking viral entry, giving no evidence that malaria exposure protects against SARS-CoV-2 infection through antibody-mediated viral neutralization. Though we saw that people in the 9 to < 18 years quartile had higher IgG and IgM reactivity than other age groups, this may have been confounded by the samples that were included. The 9 to < 18 age group had substantially more samples from infected subjects (which show higher IgG and IgM reactivity) than the other age groups (72% for 9 to < 18 group compared to 23%, 32%, and 22% for the < 9, 18–32.5, and 32.5–81 age groups, respectively).

These results are consistent with recent studies testing cross-reactivity of serological assays; two SARS-CoV-2 serological assays targeting the Nucleocapsid protein had cross-reactivity among pre-pandemic samples from Nigeria with higher levels of malaria antibodies^14^. High rates of false positives were also seen in pre-pandemic samples using commercially available ELISAs from Benin^12^, Ghana, and Nigeria^13^. Studies also showed cross reactivity was associated with *Plasmodium* parasite load (though rates of acute malaria infection was not significantly different)^12^ and past malaria exposure as measured by IgG directed against malarial antigens^14^. Our discovery that the molecular target of malaria-exposed samples is a carbohydrate rather than linear or conformational protein epitope, and that treatment with sialic acid does not influence anti-SARS-CoV-2 antibody convalescent sera reactivity, offers potential solutions for circumventing the challenge of cross-reactivity. Mutational analysis targeting known glycosylation sites in SARS-CoV-2 Spike protein impacted infectivity and antigenicity, with most glycosylation site mutants becoming less infective and more susceptible to neutralizing antibodies^33^. However, despite the emergence of mutations in SARS-CoV-2 resulting in new variants, notably there not a single mutation in N-linked glycosylation sites have been observed in the major variants of concern (VOC)^34^, and further for some VOC, mutations introduce additional potential glycosylation sites which are hypothesized to have impacts an antigen shielding^35^. These findings not only emphasize the importance of glycosylation in viral evolution, but also indicate that even if recombinant S1 proteins were expressed from COVID-19 variants of concern and incorporated into serological assays, the unmodified glycan profiles would likely continue to contribute to cross-reactive antibodies in malaria endemic areas.

Steinhardt et al. previously incorporated a urea wash into their assay to reduce non-specific binding, and we incorporated this approach into our study to measure its potential^14^. With a 4 M urea wash step, malaria samples from Steinhardt et al. had a median 31.0% avidity index, which was comparable with our avidity index (median 38.2% for malaria samples). However, Steinhardt et al. found that substantially more false positive samples became negative with 4 M urea wash (86% of 37 borderline/positive samples vs. 58% of 12 positive samples in this study). Among a subset of 20 true negative malaria exposed samples, we found that 60% were positive by S1 ELISA, 25% were positive in the avidity assay, and only 5% were positive by Hybrid DABA. These findings indicate that incorporating a urea wash, or an alternative assay to reduce non-specific binding such as the Hybrid DABA, can be effective methods to increase the specificity of SARS-CoV-2 serological tests in malaria endemic areas. Methods to increase the specificity could allow serological surveillance tools to more accurately estimate population-level SARS-CoV-2 exposure.

Our study has some limitations. Although this study benefits from the inclusion of 11 different cohorts spanning 8 countries, the parent studies of each of these cohorts had differences in inclusion criteria and sample collection that could make comparisons between cohorts difficult. Additionally, not all studies tested for malaria using molecular techniques, raising the possibility of misclassification for individuals with asymptomatic infection.

Because we included samples from available parent studies, parts of the world were underrepresented geographically, and subjects skewed young and included few non-*falciparum* malaria infections. Future studies should attempt to enroll more subjects outside of sub-Saharan Africa, more older subjects, and more subjects with non-*falciparum* malaria. Identifying whether additional malaria species, such as *P. vivax*, are associated with cross-reactivity to SARS-CoV-2 diagnostics could determine geographic regions at risk of false positive SARS-CoV-2 diagnostic tests. Additionally, while we were able to identify subjects with acute malaria infection, we did not know their history of malaria infection, and cannot exclude the possibility that serologic responses to the SARS-CoV-2 antibody test is associated with repeated malaria exposure rather than acute infection. However, many subjects with high serologic responses were in the cohort from Thiès, Senegal, an area of very low malaria transmission, where subjects were unlikely to have had many previous malaria infections. Lastly, while cross-reactive samples were not neutralizing by blocking viral entry, we did not test whether reactivity could be protective through other mechanisms such as opsonization or T-cell mediated immune responses.

These findings provide evidence of a target and mechanism for cross-reactivity and have implications for the global roll-out of serological surveillance tools reactive to SARS-CoV-2 Spike protein. Serological surveys remain critical tools in understanding disease burden; however, cross-reactivity in malaria-endemic regions, and especially in subjects with acute malaria, could lead to false positive antibody tests for individuals and overestimate population-level exposure. Although results of SARS-CoV-2 antibody tests are intended to determine exposure to SARS-CoV-2 and have not been validated to determine immunity to COVID-19, false positive results could cause an individual to underestimate their risk of future infection. At a population level, overestimates of prior SARS-CoV-2 exposure could lead to overestimates of immunity. If serological surveillance overestimates the proportion of people who have been exposed to SARS-CoV-2, then surveillance may underestimate risk of continued spread of SARS-CoV-2. Therefore, false positive antibody tests that result in overestimates of exposure could lead to predictions that underestimate severity of subsequent SARS-CoV-2 infections at a population level. Diagnostic tools for serosurveillance may benefit from using proteins lacking sialic acid to minimize cross reactivity to heavily sialated domains. Additionally, multiplexed approaches that require positive responses at multiple SARS-CoV-2 antigen targets may circumvent the challenge of false positivity due to a single protein.

## Conclusions

Cross-reactive diagnostic tools may have limited utility for serological surveillance when predicting future spread and severity of COVID-19 in areas where incidence of COVID-19 is low relative to malaria. High rates of false positives could preclude using serological tests as correlates of exposure in malaria-endemic areas, hampering policy decision making with respect to risk stratification and implementation of non-pharmaceutical interventions. These results further highlight the need to validate diagnostics in populations with different disease exposures and optimize such diagnostics so that serological surveillance tools can accurately track SARS-CoV-2 exposure. Our results identify a molecular target for cross-reactive antibodies that could potentially be exploited to improve specificity of serologic tests.

## Supplementary Information

Supplementary Information.

## Data Availability

Data associated with this manuscript can be found at: https://doi.org/10.5061/dryad.w6m905qpj.
