# Supplementary material for: Plasmodium infection is associated with cross-reactive antibodies to carbohydrate epitopes on the SARS-CoV-2 Spike protein
Source: Sci Rep. 2022 Dec 22;12:22175. doi: 10.1038/s41598-022-26709-7 (PMC9778468; doi:10.1038/s41598-022-26709-7)
Supplement: Supplementary file 1 — Supplementary Information. [file 41598_2022_26709_MOESM1_ESM.pdf]

Supplementary Information for

Plasmodium infection is associated with cross-reactive antibodies to carbohydrate epitopes on the SARS-CoV-2 Spike protein

Sarah Lapidus, Feimei Liu, Arnau Casanovas-Massana, Yile Dai, John D. Huck, Carolina Lucas, Jon Klein, Renata B. Filler, Madison S. Strine, Mouhamad Sy, Awa B. Deme, Aida S. Badiane, Baba Dieye, Ibrahima Mbaye Ndiaye, Younous Diedhiou, Amadou Moctar Mbaye, Cheikh Tidiane Diagne, Inés Vigan-Womas, Alassane Mbengue, Bacary D. Sadio, Moussa M. Diagne, Adam J. Moore, Khadidiatou Mangou, Fatoumata Diallo, Seynabou D. Sene, Mariama N. Pouye, Rokhaya Faye, Babacar Diouf, Nivison Nery Jr, Federico Costa, Mitermayer G. Reis, M. Catherine Muenker, Daniel Z. Hodson, Yannick Mbarga, Ben Z. Katz, Jason R. Andrews, Melissa Campbell, Ariktha Srivathsan, Kathy Kamath, Elisabeth Baum-Jones, Ousmane Faye, Amadou Alpha Sall, Juan Carlos Quintero Vélez, Michael Cappello, Michael Wilson, Choukri Ben-Mamoun, Richard Tedder, Myra McClure, Peter Cherepanov, Fabrice A. Somé, Roch K. Dabiré, Carole Else Eboumbou Moukoko, Jean Bosco Ouédraogo, Yap Boum II, John Shon, Daouda Ndiaye, Adam Wisnewski, Sunil Parikh, Akiko Iwasaki, Craig B. Wilen, Albert I. Ko, Aaron M. Ring, Amy K. Bei

Amy Kristine Bei.
E-mail: amy.bei@yale.edu

This PDF file includes:

Supplemental Methods
Figs. S1 to S3
Table S1

Supplemental Methods

Cohorts and Sample Collection. CAM: Samples from Douala, Cameroon were collected from patients aged 6 months or older in July to November 2018 presenting to the Emergency Department of Douala Military Hospital with fever, history of fever in previous three days, or suspected malaria. Dried blood spots of 150 µL were collected on Whatman™ 3MM chromatography paper, transported at ambient temperature, and then stored at -20°. Malaria infection and speciation was determined by extracting DNA from dried blood spots (DBS) using Chelex and amplifying DNA with *Plasmodium* genus primers in a Polymerase Chain Reaction (PCR), followed by a second round of PCR with specific species primers of 18S small subunit ribosomal DNA for *P. falciparum, vivax, malariae, and ovale*, as previously described.(1)

SEN1: Samples from Kédougou, Senegal, located in Southeastern Senegal 710 km from Dakar, were collected in July 2019 as part of ongoing surveillance conducted by Institut Pasteur de Dakar investigating causes of febrile illness. Patients were recruited from five clinics in Kédougou, Senegal. Eligibility criteria for the main study was the presence of a fever (temperature greater than or equal to 38°C) and/or a fever in the past 24 hours.(2) Positivity for *P. falciparum* was determined based on a *P. falciparum*-specific HRP2/3 rapid diagnostic test. A venous blood sample of 5ml in a EDTA vacutainer was obtained from consenting, enrolled patients and transported at room temperature from the clinic to the field lab for processing; no more than 6 hours between draw and processing. Thin and thick blood smears were made for each sample to confirm monogenomic infection with *P. falciparum* by microscopy.

SEN2: Samples from the low-transmission area of Thiès, Senegal (located about 70 km West of Dakar) come from patients who presented through passive case detection at the Service de Lutte Anti Parasitaire (SLAP) clinic in 2015 to 2017 with malaria-like symptoms, tested positive to a malaria Pfhrp2 antigen rapid diagnostic test, and had positive microscopy for *P. falciparum* monogenomic infection.(3) Participants had a mean parasitemia of 0.77% (range 0.03% to 4.89%).

BUR1: Samples from children under five in the high-transmission, rural area of Bama, Burkina Faso were collected during cross-sectional surveys in July and August 2017.(4) Children under 5 provided blood spears and dried blood spots before and after seasonal malaria chemoprevention (SMC). Malaria infection and speciation were determined through PCR from dried blood spots as previously described.(1) Capillary whole blood samples were collected 7-8 days after SMC administration. Blood was centrifuged at 2000xg for 10 min and plasma was collected and stored at -80°C.

GHA: Samples from Ghana were collected in July 2007 and June 2010. In July 2007, four communities in central Ghana were surveyed that were suspected to be endemic for hookworm.(5) The study team randomly selected households to participate, from which up to 2 adults and 3 children were enrolled. Participants submitted approximately 2 mL blood by venipuncture. Two drops of whole blood at the time of collection were used to create thick and thin smears to determine malaria infection and species of infection. The remaining blood was separated by centrifugation and plasma was stored at -80°C.

In June 2010, participants from 16 schools on a 90-km stretch of highway north of Kintampo with height for age (HAZ) of HAZ $\leq$ -1.80 or HAZ $\geq$ -0.10 were invited to participate.(6) Blood from participants were tested for malaria with a malaria rapid diagnostic test kit (First Response Malaria Ag HRP-2; Premier Medical Corporation Ltd., Watchung, NJ). Among participants who tested positive via the rapid diagnostic test, species of malaria infection was determined by microscopy. All participants were asymptomatic for malaria at the time of sample collection.

BUR2: Participants were enrolled in a cross-sectional study (NIAID R21AI097695) from late October 2016 through February 2017 in the area of Vallée de Kou (Bama) in southwestern Burkina Faso, 25 km from the city of Bobo-Dioulasso. Malaria is seasonally intense, with the dry season typically beginning in November and lasting through May. Participants were all asymptomatic healthy adults of Mossi or Fulani ethnicity, with negative malaria rapid diagnostic tests and blood smear at the time of sampling, as well as negative HIV rapid testing. Only a single adult from a household was enrolled. Plasma was collected from venous samples, stored in liquid nitrogen after processing, and shipped to Yale by dry shipper in February 2018.

COL1: Participants were surveyed in a cross-sectional study on rickettsiae infection from November 2015 to January 2016 in nine hamlets located in Alto de Mulatos in the municipality of Turbo and Las Changas in the municipality of Necoclí in in northwestern Colombia.(7) Residents of all ages were eligible for participation. Households were randomly selected proportional to the number of households in urban and rural areas, and all members of the household were eligible for inclusion. Serum was collected from all participants and stored at -20°C or -80°C. This region is one of the most affected by *P. vivax* malaria in Colombia. *P. falciparum* is also endemic in this region.

COL2: Participants were enrolled in a cohort study aimed to study rickettsia infection in Uramita, Colombia from August to September 2016. Participants were selected from households in 10 neighborhoods in the urban center and one village in a rural area. Households were randomly selected proportional to the number of households, and all members of the household were eligible for inclusion. Serum was collected from all participants and stored at -20°C or -80°C. The municipality of Uramita is not considered endemic for malaria transmission.

BRA: A prospective cohort among urban slum residents of Pau da Lima, in northwestern Salvador, Brazil was tested for serologic evidence of *Leptospira* infections in January to November 2010. This study site is density populated, has low median household per capita income, and a majority of inhabitants do not legally own their domiciles.(8) Households were randomly selected for inclusion. Subjects were eligible for inclusion if they slept at least 3 nights per week in a selected household. Eligible participants provided serum samples in 2010. Malaria is not endemic in this site in Brazil.

NEP: This cohort is comprised of subjects who presented to four peri-urban and rural health facilities in Kavrepalanchok and Dolakha, Nepal for a study on enteric fever diagnosis from August 2013 to June 2016.(9) Subjects had a self-reported >72-hour history of fever, had to be at least 12 months old, and provided a venous blood sample. Although malaria isn't completely eliminated from this area, there is very little malaria transmission in the area and subjects are unlikely to have had

malaria.

EBV: This cohort contains students who developed infectious mononucleosis caused by Epstein-Barr virus (EBV) during the course of follow-up.(10) A cohort of college students ages 18 to 20 had serum collected at Northwestern University Health Center contributed serum samples in February 2015 through October 2018 from 2 timepoints each within 2 years of diagnosis with EBV. The first timepoint was generally within 6 weeks of the diagnosis of mono (range 6 days before to 5 months after) and the second timepoint was generally 6 months following the diagnosis of mono (range 5 – 24 months).

HCW pos: The cohort of health care worker (HCW) positive for SARS-CoV-2 were prospectively followed as part of the Yale Implementing Medical and Public Health Action Against Coronavirus CT (IMPACT) study and tested positive for SARS-CoV-2 during follow-up. This cohort consisted of 13 HCWs who had serum sampled between 15 and 40 days after a positive SARS-CoV-2 diagnosis by RT-qPCR. HCWs tested positive between April 10, 2020 and December 15, 2020.

HCW neg: The cohort of health care worker (HCW) negative for SARS-CoV-2 were prospectively followed as part of the Yale Implementing Medical and Public Health Action Against Coronavirus CT (IMPACT) study. This cohort includes 80 HCWs who contributed serum between April 13, 2020 and April 23, 2020 and who had tested negative for SARS-CoV-2 infection by RT-qPCR since the start of the study. Results from this cohort were used to determine cutoffs for S1 subunit IgG and IgM positivity.

COVID-19: The cohort was made up of patients admitted to Yale New Haven Hospital with COVID-19 who had blood samples collected between April 14, 2020 and June 19, 2020. All patients had been hospitalized for at least 14 days at the time of sample collection. Sera from this cohort was tested with the S1 subunit ELISA, S1 subunit ELISA with urea wash, S1 subunit ELISA treated with neuraminidase, and the Hybrid DABA immunoassay (Kalon Bio).

YNHH: From a patient diagnosed with *P. vivax* treated at Yale New Haven Hospital in Connecticut.

Viral neutralization assays: SARS-CoV-2 virus. To determine if antibodies in patient serum was protective against wild-type (WT) SARS-CoV-2 virus by neutralization, 10 SEN2 cohort samples were selected with a range of SARS-CoV-2 S1 Spike IgG levels, but mostly including samples with high IgG, plus negative controls. Neutralization assays were conducted as previously described.(11) Serum was diluted from 1:3 to 1:2430 at sixfold serial dilutions and incubated with WT SARS-CoV-2 virus for 1 hr, and then used to infect Vero E6 cells for 1 hr. The serum/virus mixture was removed, and cells were incubated and stained for visualization of plaques at 40 hrs post-infection.

References

1. G Snounou, S Viriyakosol, W Jarra, S Thaithong, K Brown, Identification of the four human malaria parasite species in field samples by the polymerase chain reaction and detection of a high prevalence of mixed infections. *Mol. Biochem. Parasitol.* **58**, 283–292 (1993).
2. AJ Moore, et al., Assessing the functional impact of PfRh5 genetic diversity on ex vivo erythrocyte invasion inhibition. *Sci. Reports* **11**, 2225 (2021).
3. M Sy, et al., Plasmodium falciparum genomic surveillance reveals spatial and temporal trends, association of genetic and physical distance, and household clustering. *Sci Rep* **12**, 938 (2022).
4. FA Somé, et al., Investigating selected host and parasite factors potentially impacting upon seasonal malaria chemoprevention in bama, burkina faso. *Malar. J.* **19**, 238 (2020).
5. D Humphries, et al., Epidemiology of hookworm infection in kintampo north municipality, ghana: Patterns of malaria coinfection, anemia, and albendazole treatment failure. *The Am. J. Trop. Medicine Hyg.* **84**, 792–800 (2011).
6. D Humphries, et al., Hookworm infection among school age children in kintampo north municipality, ghana: nutritional risk factors and response to albendazole treatment. *The Am. J. Trop. Medicine Hyg.* **89**, 540–548 (2013).
7. JC Quintero V., et al., Eco-epidemiological analysis of rickettsial seropositivity in rural areas of colombia: A multilevel approach. *PLOS Neglected Trop. Dis.* **11**, e0005892 (2017).
8. JE Hagan, et al., Spatiotemporal determinants of urban leptospirosis transmission: Four-year prospective cohort study of slum residents in brazil. *PLoS Neglected Trop. Dis.* **10** (2016).
9. JR Andrews, et al., High rates of enteric fever diagnosis and lower burden of culture-confirmed disease in peri-urban and rural nepal. *The J. Infect. Dis.* **218**, S214–S221 (2018).
10. BZ Katz, et al., A validated scale for assessing the severity of acute infectious mononucleosis. *The J. Pediatr.* **209**, 130–133 (2019).
11. C Lucas, et al., Delayed production of neutralizing antibodies correlates with fatal covid-19. *Nat. Medicine* **27**, 1178–1186 (2021).

			Malaria exposure status							
Cohort	Country	Region	Symptomatic (%)	Asymptomatic (%)	Endemic uninfected (%)	Non-endemic (%)	Male (%)	Mean age, years (min, max)	Dates of collection	# Subjects
CAM	Cameroon	Douala	8 (42.1%)	-	11 (57.9%)	-	11 (52.4%)	26.2 (2, 64)	July-Nov 2018	19
SEN1	Senegal	Kédougou	60 (50%)	-	60 (50%)	-	67 (55.8%)	22 (1, 74)	July 2019	120
SEN2	Senegal	Thiès	67 (100%)	-	-	-	67 (100%)	10.9 (5, 16)	2015-2017	67
BUR1	Burkina Faso	Bama	-	13 (14.8%)	75 (85.2%)	-	11 (52.4%) *	2.7 (0.5,4)	July-Aug 2017	88
GHA	Ghana	Kintampo	-	29 (64.4%)	16 (35.5%)	-	17 (38.6%) **	15.1 (3, 70) **	July 2007 and June 2010	45
BUR2	Burkina Faso	Bama	-	-	25 (100%)	-	14 (60.9%) ***	32.6 (21, 43) ***	Oct 2016-Feb 2017	25
COL1	Colombia	Urabá	-	-	61 (100%)	-	24 (39.3%)	31 (5, 80)	Nov 2015-Jan 2016	61
COL2	Colombia	Uramita	-	-	-	27 (100%)	6 (22.2%)	38 (5, 70)	Aug-Sept 2016	27
BRA	Brazil	Salvador	-	-	-	80 (100%)	30 (37.5%)	30.6 (5, 71)	Jan-Nov 2010	80
NEP	Nepal	Kavrepalanchok and Dolakha	-	-	-	71 (100%)	31 (44.3%) ****	36.2 (4, 80) ****	Aug 2013-June 2016	71
EBV	USA	Illinois	-	-	-	14 (100%)	5 (35.7%)	18.7 (18, 20)	Feb 2015-Oct 2018	14
Total			135 (21.9%)	42 (6.8%)	248 (40.2%)	192 (31.1%)	282 (51.6%)	22.2 (0.5, 80.6)	July 2007-July 2019	617

*In BUR1, data on sex was available for 21 of 88 subjects
**GHA had 1 subject with unknown age and sex
*** BUR2 had unknown sex and age for 2 subjects
****NEP had unknown sex and age for 1 subject.

Table S1. Patient Demographics

Detailed demographics for all eleven cohorts outside of YNHH included in the study are presented here.

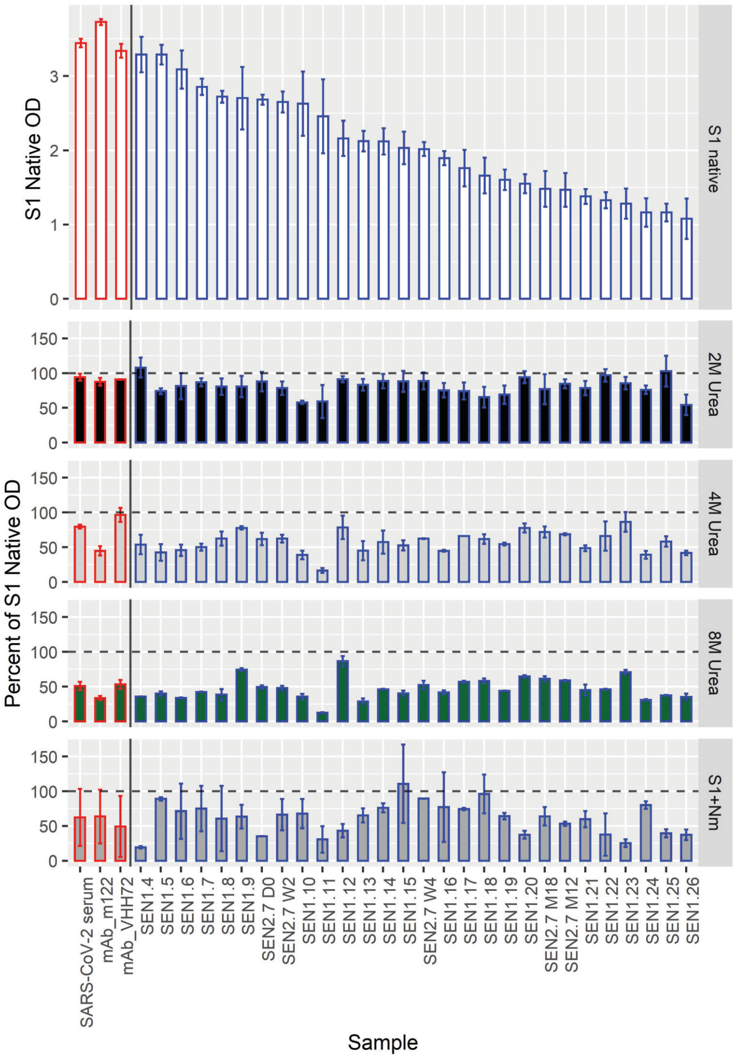

Fig. S1. Effect of different urea concentrations on malaria samples Among 28 malaria exposed samples, samples washed with 2M urea was reduced to an average of 80.9% of S1 native samples, while pooled convalescent serum was reduced to 94.0% of S1 native pooled convalescent serum. Samples washed with 4M urea was reduced to an average of 56.9% of S1 native samples, while pooled convalescent serum was reduced to 79.6% of S1 native pooled convalescent serum. Samples washed with 8M urea was reduced to an average of 46.9% of S1 native samples, while pooled convalescent serum was reduced to 50.9% of S1 native pooled convalescent serum. For comparison, S1 protein treated with neuraminidase was reduced to an average of 61.0% of S1 native samples, while pooled convalescent serum was reduced to 62.4% of S1 native pooled convalescent serum. 4M urea was determined to be the most effective urea concentration to reduce reactivity of malaria samples while having a smaller effect on true positive convalescent sera.

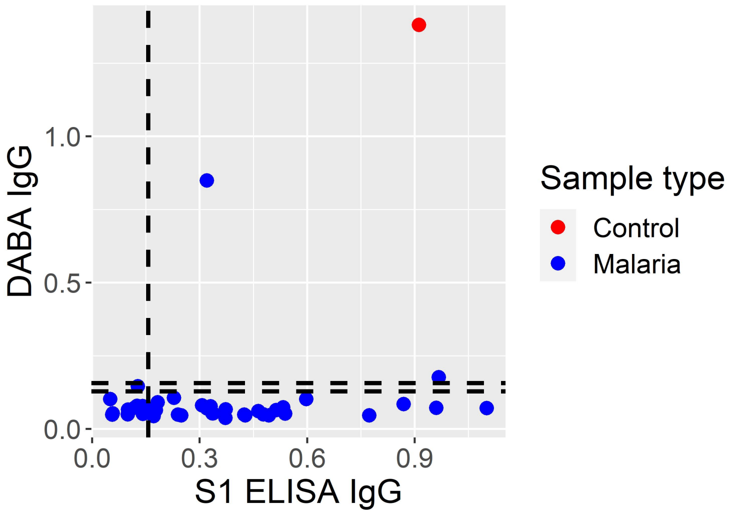

Fig. S2. S1 ELISA and Hybrid DABA for malaria samples Scatterplot showing values of S1 subunit ELISA vs. Hybrid DABA, a method of increasing specificity for antibody reactivity. 41 malaria positive and malaria exposed samples (in blue) were tested, along with a positive control (pooled convalescent serum from SARS-CoV-2 patients, in red). For S1 ELISA, dashed lines represent cutoffs for positivity, and for DABA, lower and upper dashed lines represent cutoff for negativity and positivity, respectively (values between lines are equivocal). Of 41 malaria positive and malaria exposed samples, 30 (73.2%) tested positive by S1 subunit ELISA, and 2 (4.9%) were positive and 1 (2.4%) was equivocal by Hybrid DABA.

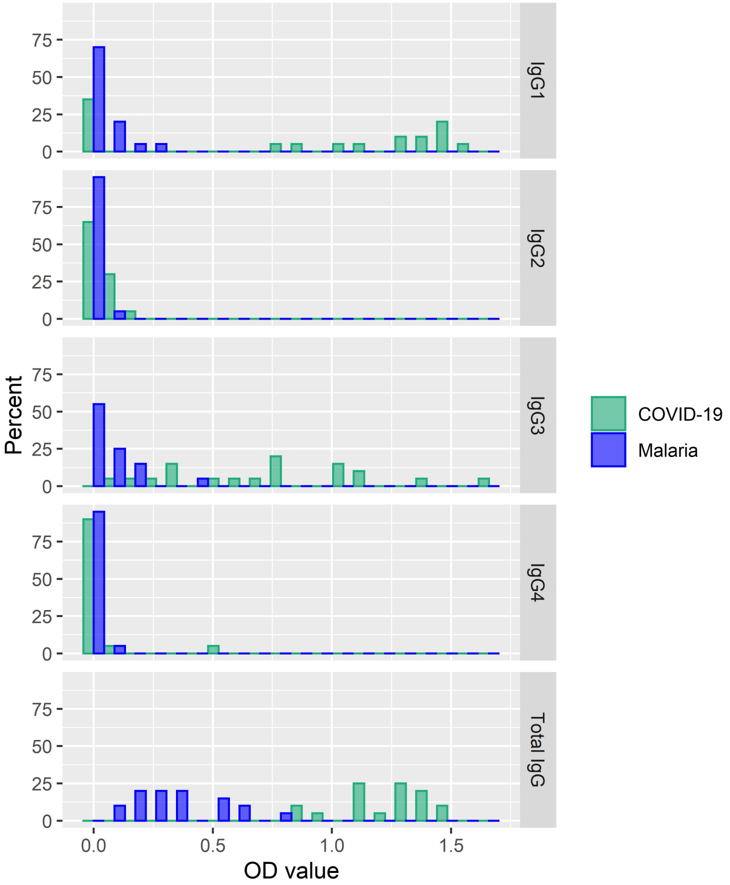

Fig. S3. IgG subclasses for COVID-19 and malaria positive samples IgG subclass values for 20 COVID-19 samples (green) and 20 malaria samples (blue). Median IgG OD values for COVID-19 samples and malaria samples were 1.09 and 0.031 for IgG1, 0.033 and 0.021 for IgG2, 0.753 and 0.043 for IgG3, and 0.016 and 0.008 for IgG4.
